# Innovative Cellular Insulation Barrier on the Basis of Voronoi Tessellation—Influence of Internal Structure Optimization on Thermal Performance

**DOI:** 10.3390/ma17071578

**Published:** 2024-03-29

**Authors:** Beata Anwajler, Sara Zielińska, Anna Witek-Krowiak

**Affiliations:** 1 Faculty of Mechanical and Power Engineering, Wroclaw University of Science and Technology, 27 Wybrzeze Wyspianskiego Street, 50-370 Wroclaw, Poland; beata.anwajler@pwr.edu.pl; 2 Faculty of Information and Communication Technology, Wroclaw University of Science and Technology, 27 Wybrzeze Wyspianskiego Street, 50-370 Wroclaw, Poland; 253780@student.pwr.edu.pl; 3 Faculty of Chemistry, Wroclaw University of Science and Technology, 27 Wybrzeze Wyspianskiego Street, 50-370 Wroclaw, Poland

**Keywords:** voronoi tessellation, cellular composites, emissivity, thermal conductivity, coefficient of heat transfer, 3D printing, additive manufacturing, thermal insulation

## Abstract

The optimization of structure and thermal properties in 3D-printed insulation materials remains an underexplored area in the literature. This study aims to address this gap by investigating the impact of 3D printing on the thermal properties of manufactured cellular composites. The materials studied were closed-cell foams with a complex cell structure based on the Voronoi cell model, manufactured using incremental technology (3D printing). The influence of the cellular structure of the composite, the type of material used, and the number of layers in the composite structure on its thermal properties, i.e., thermal conductivity coefficient, thermal resistance, and coefficient of heat transfer, was analyzed. Samples of different types of thermosetting resins, characterized by different values of emissivity coefficient, were analyzed. It was shown that both the type of material, the number of layers of the composite, and the number of pores in its structure significantly affect its thermal insulating properties. Thermal conductivity and permeability depended on the number of layers and decreased up to 30% as the number of layers increased from one to four, while thermal resistance increased to 35%. The results indicate that material structure is key in regulating thermal conduction. Controlling the number of cells in a given volume of composite (and thus the size of the air cells) and the number of layers in the composite can be an effective tool in designing materials with high insulation performance. Among the prototype composites produced, the best thermal performance was that of the metalized four-layer cellular composites (λ = 0.035 ± 0.002 W/m·K, R_c_ = 1.15 ± 0.02 K·m^2^/W, U = 0.76 ± 0.01 W/m^2^·K).

## 1. Introduction

The growing energy intensity of the country’s electricity system is prompting a search for new ways to reduce electricity demand. Conventional energy reserves like coal, oil, and natural gas are finite and diminishing. In addition, their extraction and combustion generate large amounts of greenhouse gases that contribute to climate change. As a result, urban development and technological progress are inextricably linked to appropriate energy management. Effective energy strategies and technological innovation play a key role in shaping a sustainable and future-oriented urban infrastructure [1,2]. Improving energy efficiency in various sectors, such as industry, transport, buildings, and households, can significantly reduce global energy consumption. One way to reduce energy losses is through effective thermal insulation. Improving the energy efficiency of buildings can bring significant economic and environmental benefits.

Besides traditional object manufacturing techniques such as casting, machining, and molding, the focus of this article primarily lies in incremental technology, commonly referred to as 3D printing. The additive process allows for rapid prototyping, and the resulting structures can be characterized by structural features that cannot be achieved by other manufacturing methods. It is possible to design and print almost every model using various techniques to create new shapes, such as melting material, curing resin, or laser powder sintering. The incremental method involves applying successive layers of material to form the finished object. While such methods have been utilized for centuries, such as in construction, where layers of brick and mortar are applied, today, incremental techniques have evolved into slightly different forms. The incremental model creation method is based on geometry generated in computer software and then manufactured on a numerically controlled machine. This process used to be referred to as 3D printing, with the machines responsible for producing the actual object called 3D printers.

**Figure 1:**
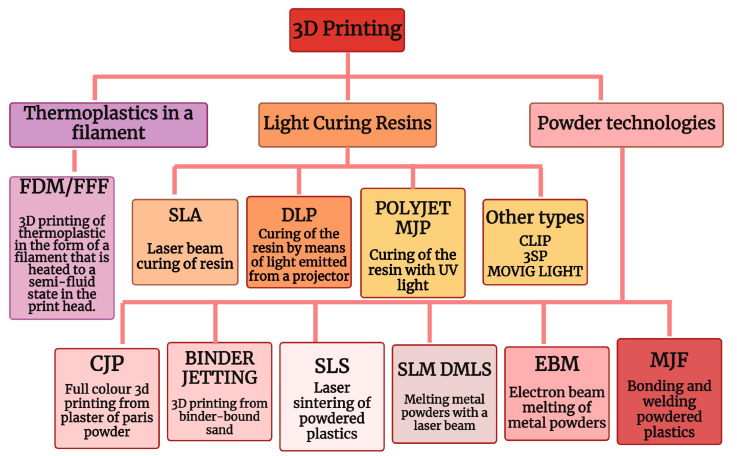
Division of incremental technologies (created in Biorender.com).

Additive manufacturing (AM), also known as 3D printing, is an innovative manufacturing technique that revolutionizes the production of three-dimensional objects. It enables the creation of intricate designs using diverse methods and raw materials, typically constructing the object layer by layer [3,4]. Three-dimensional printing can be utilized with a wide array of materials, including concrete [5], metals [6], polymers [7], ceramics [8], tissues [9], and soil [10]. AM is an oversight term that encompasses several different technologies. According to ISO/ASTM 52900:2021 [11], there are seven categories of AM processes (ISO/ASTM International, 2021). According to ISO/ASTM 52900:2021, three categories of additive manufacturing (AM) processes are central to this study: Fused Deposition Modeling (FDM), Selective Laser Sintering (SLS), and Stereolithography (SLA). SLA exemplifies a technology employing liquid raw materials, FDM demonstrates a process utilizing solid build materials such as thermoplastics, while laser powder sintering (SLS) represents a technology employing powders [12,13]. A sample breakdown of the types of additive technologies used is shown in Figure 1.

Three-dimensional printing has become the most powerful technology for rapid design, prototyping, and manufacturing [14]. Three-dimensional printing allows the creation of intricate and complex structures that can maximize surface area and improve heat transfer performance. Custom designs, such as fins and meshes, can be optimized for specific heat dissipation requirements. Complex geometries and internal structures are among the key benefits of 3D printing technology. These features make it possible to create complex and customized designs that are difficult or impossible to achieve with traditional manufacturing methods.

Examining complex geometries and internal structures in the context of 3D printing, we see that these materials possess a structure comprising a matrix, which can be solid or flexible, along with voids called pores. These porous structures can have different sizes, shapes, and arrangements, giving them different properties and applications. The matrix, the solid part of the structure, surrounds and holds the pores. The matrix material can be plastic, metal, ceramic, or any other substance. The voids (pores) are areas within the structure that are not filled with matrix material and give the porous structure its characteristic properties. The voids may be isolated or interconnected to form a network of channels. The voids can be filled with various substances such as air, phase change materials, or liquid. This saturation affects the thermal, mechanical, and conductivity properties of porous materials. Examples of applications for porous materials include thermal insulation, energy storage materials, filters, membranes, and structures for biomechanical purposes. 

**Figure 2:**
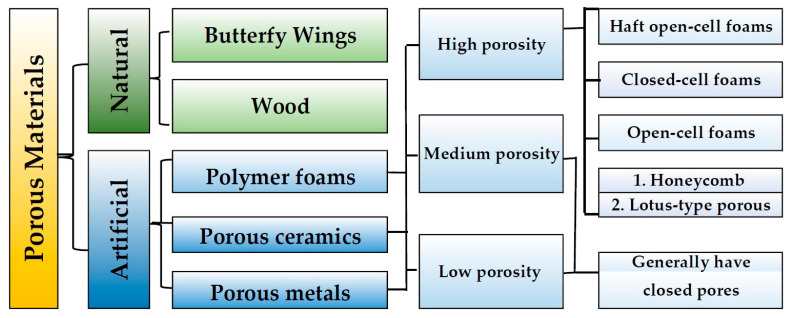
Classification of porous materials (based on [15,16,17,18]).

The classification of porous materials presented by [15,16] (Figure 2) encompasses various factors, including applications, properties, morphological parameters, materials, and manufacturing methods. 

### 1.1. Heat Flow through the Insulation—Influence of the Type of Material Used in the Printing Process

Heat flow through insulation is a very complex phenomenon. It consists of heat conduction in the gas and solid components, radiative heat transfer within the components, convection, and movement of moisture in the pores associated with its sorption and desorption. The parameter used to evaluate thermal insulation quality, known as the effective thermal conductivity coefficient (λ), is intricately influenced by variables such as pressure, temperature, the chemical composition of solids and gases, porosity, particle shape, dimensions, and numerous other factors. Insulating materials use mechanisms to reduce the contribution of each component to the total heat transfer to achieve their purpose. Reduction of conduction and convection is typically achieved by using a structure containing a large number of tiny gas-filled spaces, while reduction of radiation is achieved by using low-emissivity surfaces designed to reflect radiant heat [19,20]. A material’s emissivity (ε) is strongly associated with its surface properties. Materials have different abilities to emit infrared radiation from their surface. This property depends on the smoothness and color of the surface. Materials with dull and dark surfaces emit infrared radiation better than materials with smooth and bright surfaces. Emissivity values range from 0 (ideal reflector/mirror) to 1 (ideal emitter/black body) [21]. Note that radiance can only be considered a surface phenomenon for opaque materials, where thermal radiation is absorbed or emitted in the first few microns of the surface. In the case of transparent bodies, radiation is not a surface phenomenon, so the material’s permeability, reflectivity, and absorptivity must be considered when studying radiative properties [22]. Some materials can be considered semipermeable, semi-reflective, or semi-absorbent according to Equation (1) [20]:

1 = τ + ρ + α, (1)

where:

τ—permeability (permeability coefficient),
ρ—reflectivity (reflection coefficient),
α—absorptivity (absorption coefficient).

This work explores various resin types, including transparent, white, gray, black, and metalized, each exhibiting distinct emissivity, transmittance, reflectivity, and absorptivity properties. In addition, the materials described above have been associated with the experimental determination of the thermal properties of single, double, triple, and quadruple-layer versions of cellular composites. Heat transfer through this type of insulation, in addition to convection and conduction forces, strongly depends on selected material properties related to radiative heat transfer processes.

### 1.2. Complex Geometries for Poor Heat Transfer

To implement the idea of sustainable development, it is necessary to manage energy wisely, including reducing energy consumption and improving the efficiency of its use [23,24]. The development of innovative insulation materials can help to achieve the goals described. Cellular structures and foams are indispensable in insulation applications due to their exceptional thermal properties. With low thermal conductivity, they effectively impede heat transfer, maintaining temperature differentials. The cellular structures were formerly called honeycombs or foam materials. Honeycomb has a regular structure, such as tetragonal, pentagonal, and hexagonal. Foams, on the other hand, are characterized by irregular structures, with open and closed cells, with porosity (that refers to the ratio of void spaces within a material to its total volume, expressed as a percentage) ranging from 40% to 98% [25]. The porosity and density of these structures play a crucial role, with lower densities and higher porosities correlating with enhanced insulation by trapping more air, a poor conductor of heat. Cellular structures and foams provide versatile solutions across industries, from construction to automotive, enabling efficient thermal management and energy conservation.

Research and development of new materials suitable for 3D printing that meet thermal insulation standards could be an important area of research. In addition, 3D printing enables the realization of complex geometries that can affect energy efficiency. Traditional thermal insulation materials typically exhibit conductivity values spanning from 0.022 to 0.070 W/(m·K), making them favored options for the majority of construction projects due to their cost-effectiveness. Alongside conventional choices like expanded polystyrene (EPS) or extruded polystyrene (XPS), there is a growing trend toward alternative options such as hemp and flax, as well as highly insulating materials like vacuum insulation panels (VIP) and silica aerogels [26].

Thermal insulation materials are essential for reducing heat transfer in various applications, including building insulation, refrigeration units, and pipelines. The definition of thermal insulating material for construction purposes, as outlined in the previously withdrawn standard PN-89/B-04620 [27], identifies such materials as those with a thermal conductivity coefficient not exceeding 0.175 W/(m∙K) at 20 °C. The more recent standard PN-ISO 9229:2005 [28] recognizes thermal insulations with a thermal conductivity coefficient not exceeding 0.065 W/(m∙K), which has since been updated by the latest PN-EN ISO 9229:2020-12 [29]. Thus, it can be seen that there are increasing requirements for thermal insulation efficiency. A common feature of these materials is their complex internal structure. Most often, they are porous materials, the solid component of which is fibers or other solid substances, while the material filling the resulting pores is air or other gases with better insulating properties, such as carbon dioxide and chlorofluorocarbons. Verbeke et al. [30] highlight the pivotal role of thermal insulation materials in minimizing a building’s energy demand, particularly in regions dominated by heating requirements. Consequently, energy performance standards prescribe specific criteria for parameters like the heat transfer coefficient (U) of structural elements (e.g., walls, roofs, and floors), measured in W/(m^2^·K), or its reciprocal, the thermal resistance (R), measured in (m^2^·K)/W. The effectiveness of insulation materials can be assessed under stationary conditions by their thermal conductivity (λ), quantified in W/(m·K), or under dynamic conditions by their thermal diffusivity (D, m^2^/s) [31]. Parameter λ represents a material’s inherent ability to conduct heat across temperature gradients, and D characterizes thermal inertia, indicating the material’s capacity to buffer fluctuations in heat flow by absorbing and storing thermal energy within structural components, thereby attenuating heat transfer.

The application of Voronoi diagrams proves valuable in studying the spatial arrangement of pores within porous materials. Through this method, researchers can effectively assess and define pores’ dimensions, morphology, and spatial dispersion. Furthermore, Voronoi diagrams enable the modeling of thermal conductivity in such porous materials and facilitate the design of porous materials with desired properties, including mechanical strength and thermal conductivity. The purpose of the present research was to analyze prototype cellular materials produced using 3D printing technology. The study aimed to evaluate the thermal insulation properties of the fabricated materials based on the experimentally determined coefficient of thermal conductivity, λ, and the thermal resistance, R, for each of the materials tested. Both quantities allow us to compare different types of materials in terms of their suitability for thermal insulation. The experiments were designed to investigate the influence of cellular structure, material type, and layering on the thermal properties of the prototype cellular materials.

## 2. Materials and Methods

### 2.1. Research Material

**Figure 3:**
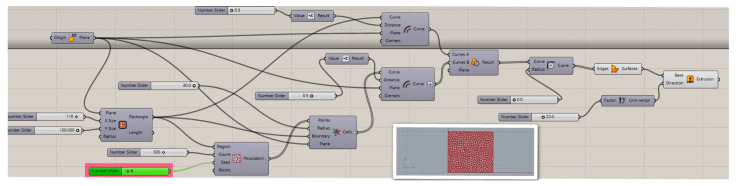
Software developed in Rhino 7 used to generate structures based on the Voronoi model (own work).

Based on a review of the literature and the previous work of one of the authors of this paper [32,33,34,35,36,37], closed-cell foams with different cell structures, different numbers of layers from 1 to 4, and different colors were selected for specific research. The choice of closed-cell foams to study the thermal properties of 3D prints is justified by several key factors, such as the potential for thermal insulation, structural diversity, and the possibility of practical application. In the Rhino 7 design software, a two-dimensional model of a closed-cell foam containing randomly distributed air voids and solid walls was generated using a Voronoi diagram using prewritten software (Figure 3). The designed composites, measuring 50 × 50 × 20 mm with a wall thickness of 0.2 mm, were then printed on an available SLS printer based on incremental selective laser sintering technology.

**Figure 4:**
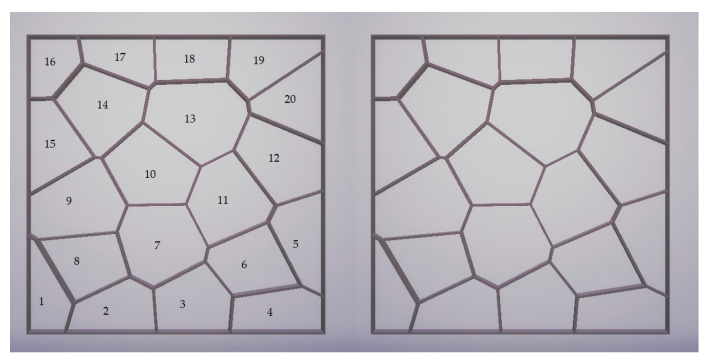
Samples of cellular composites with a pore count of 20 and a wall thickness of 0.2 mm.

**Figure 5:**
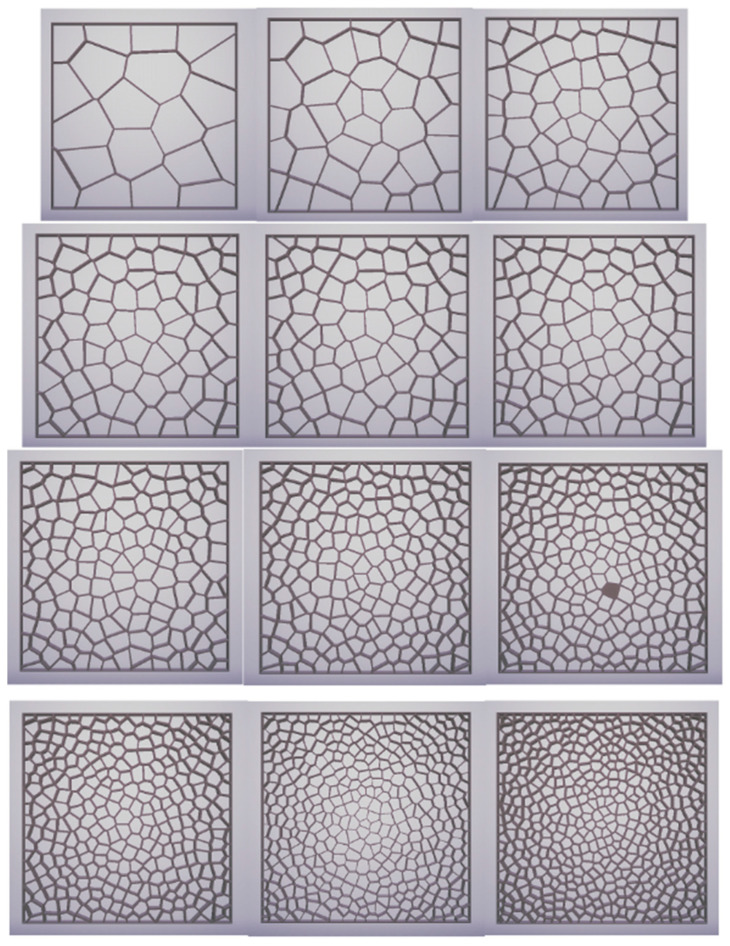
Samples of cellular composites in the following variants—number of pores, air cells at a wall thickness of 0.2 mm: 20; 40; 60; 80; 90; 100; 120; 150; 200; 300; 400; 500.

**Figure 6:**
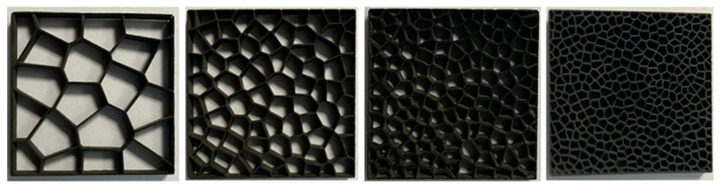
Examples of SLS print samples.

Preliminary tests were performed on SLS-printed (Figure 4, Figure 5 and Figure 6) cellular composite samples with the following variations: number of pores, air cells at 0.2 mm wall thickness: 20; 40; 60; 80; 90; 100; 120; 150; 200; 250; 300; 350; 400; 500 and a wall thickness of 0.2 mm; three-layer composite tests.

**Figure 7:**
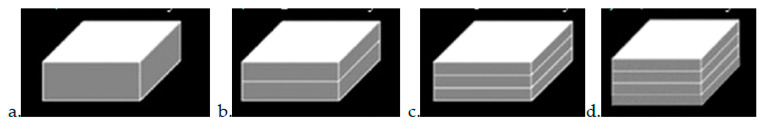
Illustration showing the number of layers in additive printed cellular composites: (**a**) 1, (**b**) 2, (**c**) 3, (**d**) 4 layers.

For the next research stage, the composite concept with the best insulating properties determined in the preliminary research, i.e., number of cells 500, wall thickness 0.2 mm, and dimensions 100 × 100 mm—single layer, double layer, triple layer, and quadruple layer—was used. Figure 7 shows a sketch of thermal partitions with different layers of thermal insulation.

**Figure 8:**
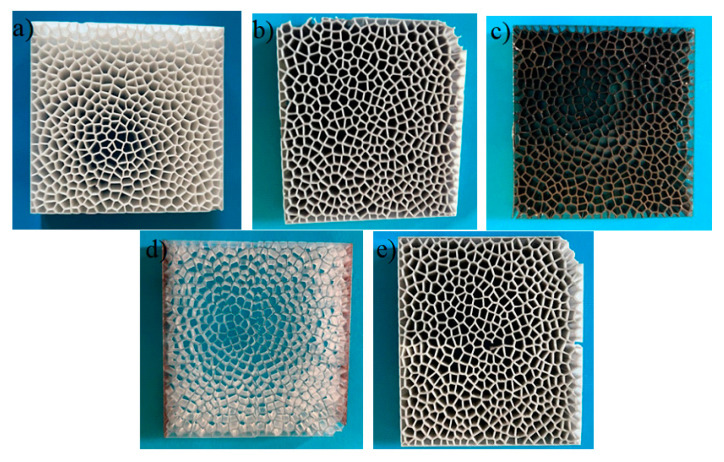
Prints of designed thermal partitions with different coloring: (**a**) white, (**b**) grey, (**c**) black, (**d**) transparent, (**e**) metalized.

From the program mentioned above created in Rhino, composite samples were generated and printed using 3D DLP—Elegoo Mars model 3 Pro printing technology, using materials (resins) with different emissivity coefficients (ε), i.e., transparent, white, gray, black, and metalized. Figure 8 summarizes prints of designed thermal baffles with different colors: transparent, white, gray, metallic, and black.

The choice of samples of different colors was dictated by the different emissivity coefficients of the composites produced. White-colored samples have a relatively low emissivity coefficient. In addition, they effectively reflect light, resulting in lower energy absorption and heat emission. Black samples have a high emissivity coefficient (about 1), which makes them effective at emitting heat. They also have low reflectance, so they absorb a lot of light. Gray samples exhibit characteristics that fall between white and black materials in terms of emissivity and reflectance values. Transparent samples are characterized by low emissivity, high light transmission efficiency, and low thermal emissivity. Metalized samples are characterized by low emissivity and efficiency in reflecting thermal radiation. By testing 3D printing foams, it is possible to assess how their insulating properties affect the thermal behavior of prints and their response to varying temperature conditions. Studying various layer thicknesses in foams provides insights into how material density influences thermal conduction. This can help determine the optimal layer thickness for the best thermal performance. A material’s color can affect its thermal properties, i.e., dark colors can absorb more thermal energy than light colors, resulting in differences in heat dissipation. Studying different foam colors helps to understand these differences and their effect on thermal stability. In addition, the choice of closed-cell foams was dictated by practical application. This type of material is used as insulation in various industries, including construction, aerospace, medical, and food. Understanding the thermal properties of 3D-printed insulation materials can have practical applications in the design of thermal envelopes. In addition, exploring new materials for 3D printing is essential to developing this technology. Closed-cell foams are one of the promising options due to their properties, and understanding their thermal behavior in the context of 3D printing is both scientifically and practically crucial for the development of this technology and can contribute to the development of more efficient and advanced applications.

### 2.2. Experimental Research

**Figure 9:**
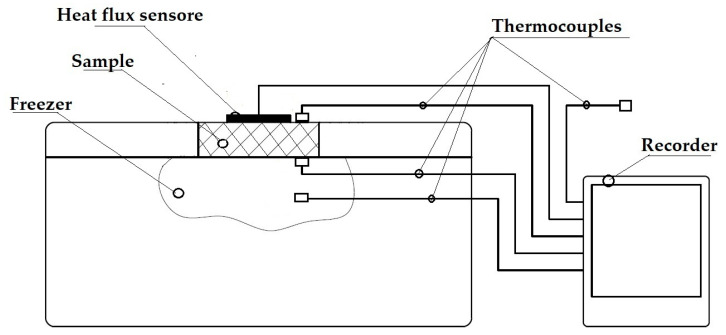
Schematic of the test stand for thermal insulation testing.

Experimental determination was conducted for each prototype composite sample, wherein the values of the coefficient of thermal conductivity, λ, the thermal resistance, R, and the coefficient of heat transfer, U, were assessed. Investigational tests were performed according to ISO 9869-1:2014 [38] on an available test stand at the Faculty of Mechanical and Power Engineering, Wroclaw University of Technology, as shown in Figure 9.

The test samples were inserted into a hole made in the lid of the Aisberg LP15 C15 freezer in a way that the bottom wall was in direct contact with the interior and the top wall was in contact with the environment. The mechanism of heat flow through sample was the difference between the ambient temperature and the temperature inside the refrigerator/freezer. The value of the heat flux density flowing through the insulation under test was measured using an FHF04SC sensor (Hukseflux Thermal Sensors B.V., Delft, The Netherlands), and the data were recorded on a recorder. During the measurements, temperatures were continuously measured and recorded on the outside wall of the sample, inside the refrigerator/freezer, and at ambient temperature. The values of the temperatures prevailing on the outer sides of the sample were assumed to be +22 °C (on the ambient side) and −20 °C (inside the refrigerator/freezer) due to the typical operating conditions of the insulating buildings, the food industry, and transportation of frozen goods. The values of thermal conductivity coefficient λ, thermal resistance R, and the heat transfer coefficient U were calculated from the measured values. The measured values were obtained after thermal equilibrium was established. The accuracy of the measuring instruments is given in Table 1.

Measurements were performed after thermal equilibrium was established. This state was considered achieved when the temperature fluctuation on the surface of the samples did not exceed 0.5 °C for 1 h during consecutive measurements. It was recorded by monitoring the stability of the measured values (U, T_g_, T_d_). In addition, each measurement performed was repeated three times to obtain more accurate results. Samples of prototype sandwich composites were tested in boxes printed in the same manner and material as the composites. Measurement uncertainties for the tested samples were 0.001~0.002 W/(m·K) for the thermal conductivity coefficient (λ) and 0.02~0.03 (m^2^·K)/W for the thermal resistance coefficient (R). 

## 3. Results

Statistical analyses were performed using tools provided by STATISTICA 13 (TIBCO Statistica, Palo Alto, CA, USA). A significance threshold of *p* ≤ 0.05 was used in accordance with common practice in thermal insulation research.

The thermal conductivity coefficient (λ) values for samples produced using 3D SLS technology varied between 0.035 and 0.067 W/(m·K), with a mean of 0.053 W/(m·K) and a standard deviation of 0.011 W/(m·K) (refer to Table 2). Approximately half of the tested samples exhibited a thermal conductivity coefficient of 0.056 W/(m·K) or lower. Regarding the thermal resistance (R), the recorded values ranged from 0.297 to 0.576 (m^2^·K)/W, with an average of 0.401 (m^2^·K)/W and a deviation of 0.095 (m^2^·K)/W. Again, roughly half of the samples displayed a thermal resistance value of 0.361 (m^2^·K)/W or less. The distribution showed high skewness and kurtosis, indicating that the majority of sample results were clustered around the mean and were relatively low.

The thermal conductivity coefficient (λ) values ranged from 0.035 to 0.057 W/(m·K), with a mean of 0.043 W/(m·K) and a standard deviation of 0.007 W/(m·K) (see Table 3). Approximately half of the tested samples yielded a thermal conductivity coefficient of 0.042 W/(m·K) or lower. Concerning the thermal resistance (R), the recorded values varied between 0.680 and 1.155 (m^2^·K)/W, with an average of 0.948 (m^2^·K)/W and a standard deviation of 0.131 (m^2^·K)/W. Similarly, about half of the samples displayed a thermal resistance value of 0.951 (m^2^·K)/W or lower. The coefficient of heat transfer (U) ranged from 0.755 to 1.152 W/(m^2^·K), with a mean of 0.908 W/(m^2^·K) and a standard deviation of 0.115 W/(m^2^·K). Roughly half of the tested samples exhibited a coefficient of heat transfer of 0.871 W/(m^2^·K) or less. The observed high skewness and kurtosis suggest that the majority of sample results were relatively low and clustered around the mean.

Subsequently, the significance of the input quantities’ impact on the output quantity was assessed. ANOVA analysis of variance was used to determine this influence. The obtained results are presented in Table 4. The significance levels (*p*-values) provided in the last column of the table, being less than 0.05, indicate a notable impact of the number of air cells in composites manufactured through 3D SLS printing technology, as well as the material type (material emissivity, ε), and the number of layers (n) in composites produced by 3D DLP printing technology, on the thermal conductivity coefficient, thermal resistance, and heat transfer coefficient of the tested materials. The influence of the number of pores, material type, and structural layer count on the thermal conductivity coefficient (λ) was established, along with an assessment of potential interactive dependencies. A similar analysis was conducted for thermal resistance (R) and heat transfer coefficient (U). 

**Figure 10:**
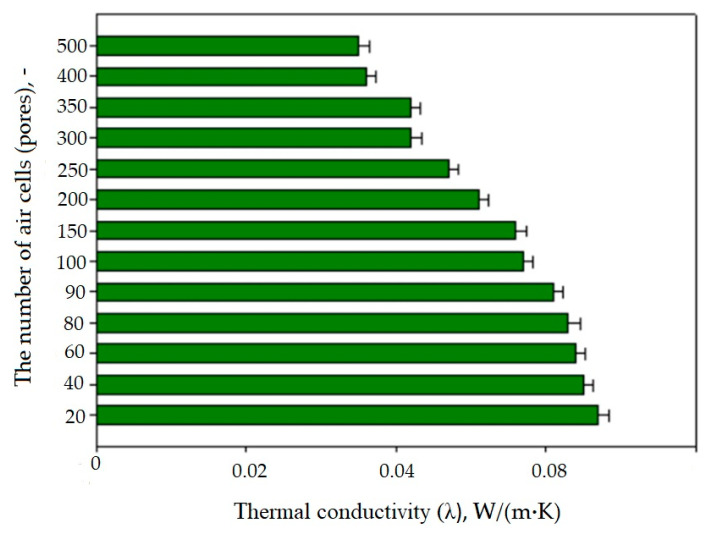
The influence of the cellular structure (the number of pores) on the value of the thermal conductivity λ of the manufactured cellular composites.

**Figure 11:**
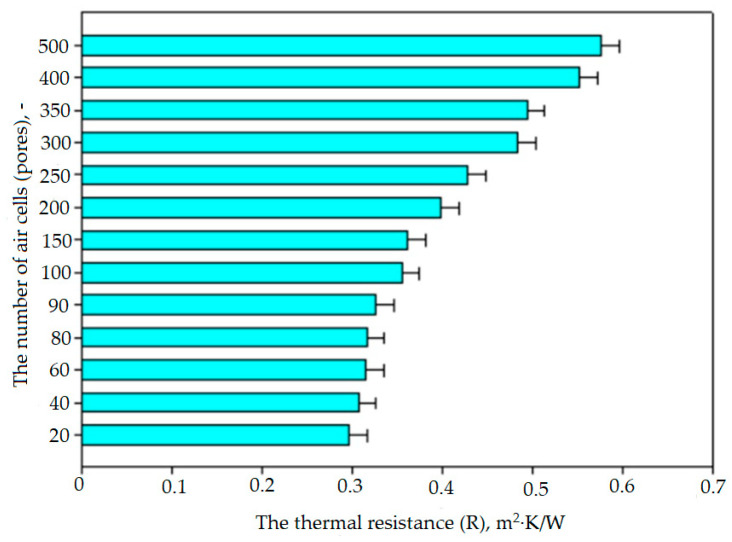
Influence of the cell structure (number of pores) on the value of the thermal resistance Rc of the produced cellular composites.

**Figure 12:**
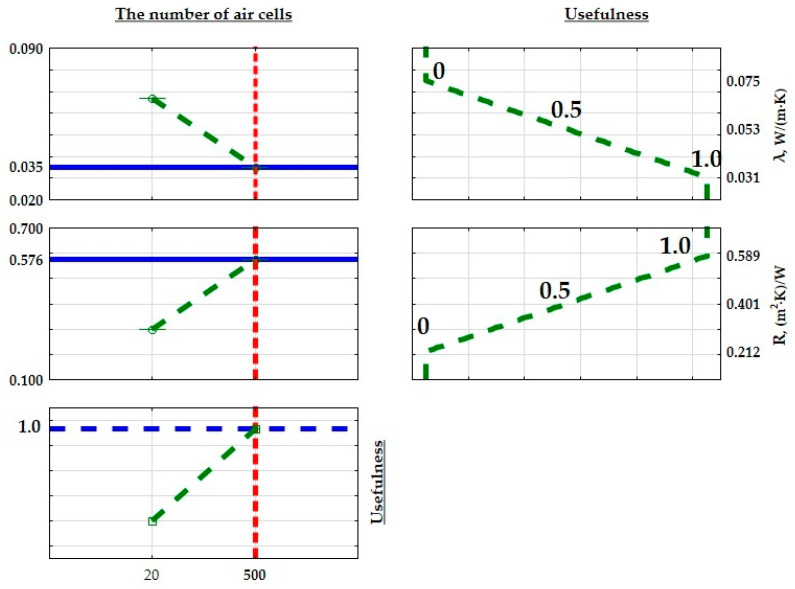
Approximate value profiles and utilities for SLS 3D-printed samples.

Figure 10, Figure 11 and Figure 12 summarize the averaged values of the coefficient thermal conductivity, λ, and the coefficient thermal resistance, R, measurements for SLS-printed three-layer materials with different numbers of pores, air cells at a wall thickness of 0.2 mm. The cellular composite samples were tested with the number of air cell variants of 20, 40, 60, 80, 90, 100, 120, 150, 200, 250, 300, 350, 400, and 500. The results show that an increase in the number of air pores in the composite structure resulted in a statistically significant decrease in the value of the thermal conductivity coefficient λ. The thermal conductivity coefficient λ values were almost half lower for the 500-pore sample than for the 20-pore sample. For example, for the prototype cellular material, the thermal conductivity coefficient λ for the 20-pore sample was 0.067 ± 0.002 W/m·K, while it was only 0.035 ± 0.002 W/m·K for the 500-pore sample.

In the case of prototype cellular materials in the studied range of the number of pores (20–500), it was shown that the higher the number of pores in a single layer of composites, the lower the value of λ and the higher the value of R resistance. The effect of the different numbers of pores, air cells at a wall thickness of 0.2 mm on the values of both studied thermal parameters (λ and R) is significant. The best thermal properties are obtained for the 500-pore variant.

**Figure 13:**
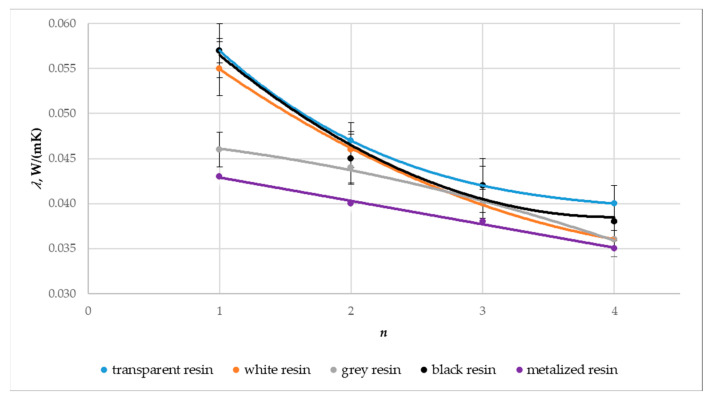
Effect on the thermal conductivity coefficient (λ) regarding the material type utilized and the composite’s layer count, denoted as n.

**Figure 14:**
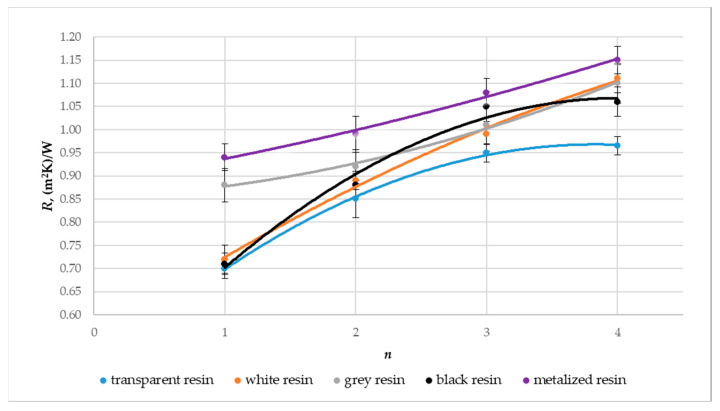
Effect on thermal resistance (R) regarding the material type utilized and the composite’s layer count, denoted as n.

**Figure 15:**
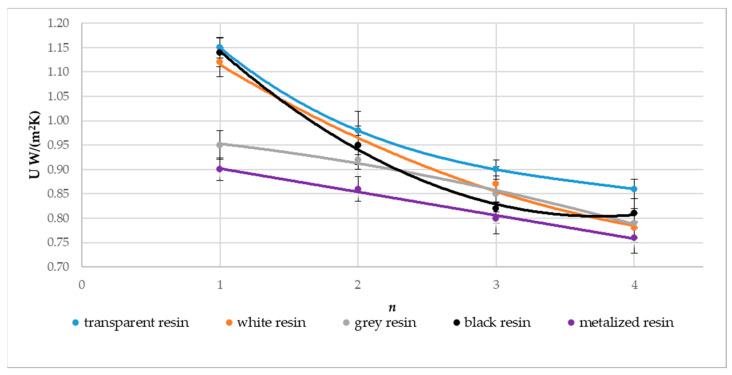
Effect on the heat transfer coefficient (U) regarding the material type utilized and the composite’s layer count, denoted as n.

Figure 13, Figure 14 and Figure 15 show a graphical comparison of the results obtained. The metalized resin samples had the lowest thermal conductivity coefficient (λ) and the heat transfer coefficient (U) and, thus, the highest thermal resistance coefficient (R) compared to the gray, black, white, and transparent resin samples. The composites made from transparent and black resin exhibited the highest thermal conductivity and, thus, the lowest thermal resistance coefficient. This indicates that the metalized resin composite made with the DLP technology had the best insulating properties. The graphs above also show the relationship between the composite’s number of layers (n) and the thermal conductivity and thermal resistance coefficient values obtained. Samples with a layer count of n = 4 demonstrated a reduced thermal conductivity coefficient, resulting in higher thermal resistance compared to samples with n = 3, n = 2, and n = 1 layers. 

**Figure 16:**
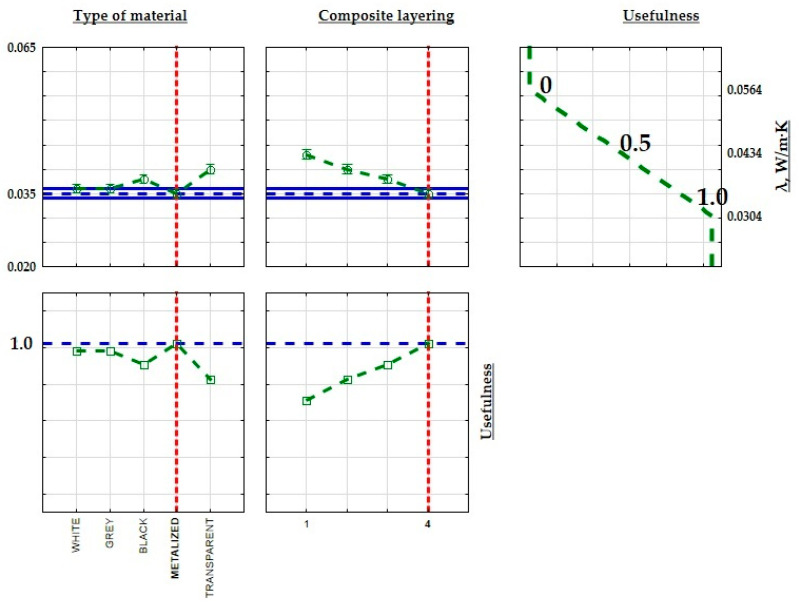
Approximate value and useful profiles of the thermal resistance coefficient (R) for 3D-printed parts produced using the SLA process.

**Figure 17:**
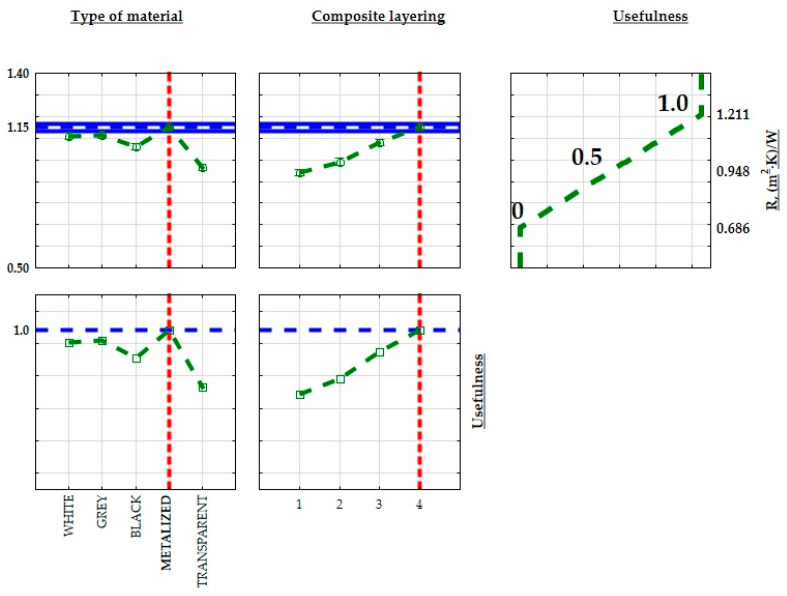
Approximate value profiles and utilities for SLM 3D-printed samples the thermal resistance coefficient (R).

**Figure 18:**
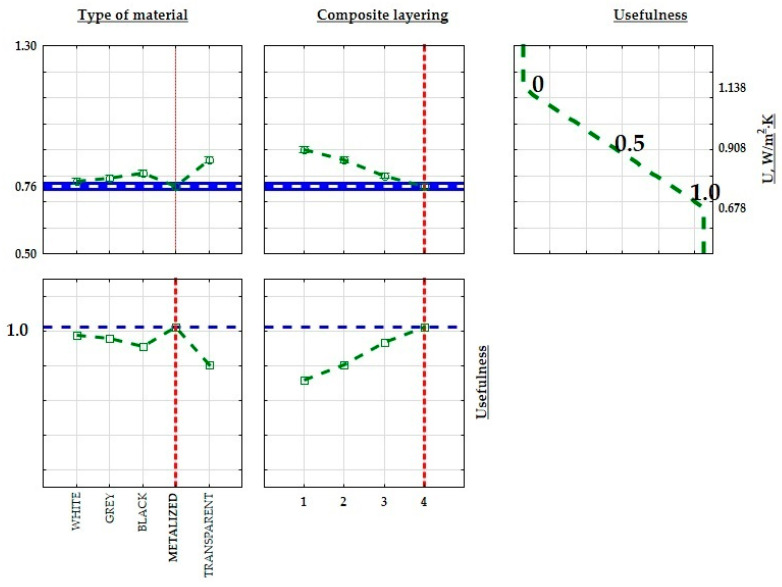
Approximate and useful heat transfer coefficient (U) profiles for 3D-printed samples produced by the SLA method.

In order to optimize the structure of the developed composites for improved thermal properties in insulation for construction and industrial applications, minimum, average, and maximum values were calculated for both thermal conductivity, thermal resistance coefficient, and heat transfer coefficient. These limits were then assigned practical values. When maximizing a parameter (max criterion), the maximum value was assigned a practical value of 1.0, while the minimum value was assigned 0. The practical values transitioned linearly between these thresholds, with the midpoint set at 0.5. Figure 16, Figure 17 and Figure 18 illustrate the extreme mean values of the thermal conductivity and thermal resistance coefficients. Given the relatively consistent values of the thermal conductivity coefficients, the lowest thermal conductivity and the highest thermal resistance and heat transfer coefficient were chosen as the criteria for identifying the optimal insulating properties of the produced composite. The material with the most favorable thermal insulation properties had the highest utility.

## 4. Discussion

**Figure 19:**
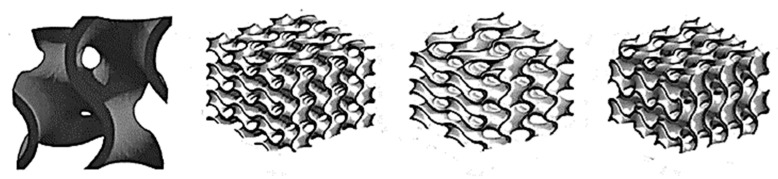
Structure of the Gyroid (based on [32]).

**Figure 20:**
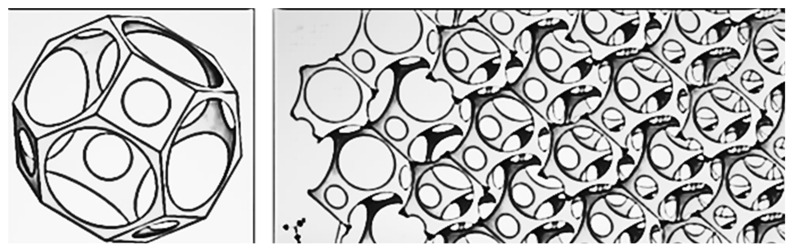
Structure of the Celvin tetrahedron (based on [33]).

The present study analyzed the effect of cellular structure, material type, and layering of cellular materials on thermal properties, i.e., thermal conductivity coefficient, thermal resistance, and thermal transmittance of composites. The number of pores within the composite structure significantly influenced the thermal properties of the produced composites. Notably, the thermal conductivity (λ) of the samples with 500 pores was approximately half that of those with 20 pores, with values of 0.035 ± 0.002 W/m·K and 0.067 ± 0.002 W/m·K, respectively. Additionally, increasing the number of layers in the structures led to improved insulation properties, with conductivity and thermal transmittance decreasing by up to 30% as the number of layers increased from one to four. In comparison, thermal resistance increased by up to 35%. The control of porosity and layering in cellular composites emerges as a valuable strategy for achieving desired thermal characteristics, offering significant enhancements in thermal performance for insulation applications in buildings, vehicles, or equipment. Moreover, the color of the composite materials played a crucial role in their thermal properties, with transparent and black samples exhibiting higher thermal conductivity (λ) compared to gray and metalized samples. Among all prototype materials tested, four-layer cellular composites with metalized color demonstrated the most favorable thermal properties, with λ = 0.035 ± 0.002 W/m·K, R_c_ = 1.15 ± 0.02 K·m^2^/W, and U = 0.76 ± 0.01 W/m^2^·K. Furthermore, all three-layer and four-layer structures met the requirements of the construction industry in terms of the heat transfer coefficient, indicating their potential suitability for use in window production. Academic work has been performed to study the thermal efficiency of such configurations designed using spatial Voronoi tessellation [39]. Researchers have studied both standard and spatial Voronoi gradient structures for thermal insulation and have investigated the influence of porosity, gradient orientation, and heat flux density on the thermal performance of structures [39]. Findings suggest that the spatial Voronoi gradient structure exhibits a lower effective thermal conductivity compared to the regular structure. Moreover, the effective thermal conductivity experienced a notable decrease with higher porosity levels. The advancement of sustainable insulation materials in modern times necessitates consideration of their environmental footprint and the sourcing of raw materials. Panda et al. [40] conducted an experimental characterization of the mechanical properties of hexagonal honeycomb cell structures printed via FDM. Meanwhile, Kam et al. [41] devised additively manufactured wood components mirroring the chemical, mechanical, and thermal attributes of natural wood, achieving λ values ranging from 0.05 to 0.085 W/(m·K). Additionally, Liu et al. [42], utilizing the FDM process, assessed the thermal conductivity of thermoplastic polyurethane samples filled with hexagonal boron nitride plates, noting a dependency on plate orientation and an increase in λ with higher filler loading. Incorporating aerogels into cementitious composites has been explored to mitigate thermal conductivity. For instance, Baghban [43] developed a rapid-setting material incorporating aerogel granules, phosphate-based cement, and fly ash, yielding a λ of 0.04 W/(m·K). By controlling the internal geometry and combinations of filler percentages, 3D printing allows both thermal conductivity optimization and lighter component creation [44,45,46], as demonstrated by experiments conducted by various researchers [32,47]. Layered composites are frequently employed [32,33,34,35], featuring a low-density core surrounded by rigid outer layers. The core material may consist of closed- or open-cell foam or periodic structures [48,49]. Islam et al. [47] investigated PLA filament thermal insulation materials using FDM printing technology. The authors showed good insulating properties as evidenced by thermal conductivity values obtained from thermal tests ranging from 0.037 W/(m⸱K) to 0.070 W/(m⸱K). De Rubeis et al. [50,51,52] investigated the insulation capacities of blocks with different internal structures printed using FDM technology from PLA filament and how these capacities are affected by filling the blocks with waste materials such as polystyrene, wood sawdust, wool, and hemp. It was shown that among all the internal structures tested, the most complex honeycomb structure provided the best insulating properties (a permeability value of 1.22 W/(m^2^⸱K)). In addition, filling the honeycomb block with waste insulation materials significantly improved its insulating properties, reducing the permeability coefficient to a value of 0.53 W/(m^2^⸱K), or by about 57%. Extensive research in printed insulation materials has been carried out by Anwajler [32,33,34,35,36,37], who studied various variants of polymer composites printed using FDM and SLS technologies (Figure 19 and Figure 20). The researcher analyzed the effects of infill structures and layering of the composites on the insulation parameters. Her research showed that the printed composites could be effectively used as insulating materials, with minimum thermal conductivity coefficients as low as 0.023 W/(m⸱K).

## 5. Conclusions

In conclusion, the study highlights the significant impact of cellular structure, material type, and layering on the thermal properties of composites, with the number of pores, air, and layers of composite playing crucial roles. Control of the number of air pores and layering offers valuable strategies for achieving desired thermal characteristics, particularly enhancing insulation properties for various applications.

The investigation into multilayer porous structures promises to revolutionize the production of energy-efficient, resilient, and eco-friendly 3D-printed insulation materials, expanding their utilization beyond the construction sector to various other industries. Existing possibilities to improve insulation materials by enhancing their properties will result in minimizing environmental impact, promoting technological progress, and developing smart adaptive solutions. Future research directions include optimizing the thermal and mechanical characteristics of 3D-printed insulation materials, exploring the incorporation of renewable or biodegradable resources, innovating multifunctional and intelligent insulation materials, refining printing processes, and extending the application of 3D-printed materials across diverse industrial domains. These recommendations have the potential to significantly increase insulation performance, sustainability, and versatility, driving progress in both construction and related sectors.

## Figures and Tables

**Table 1:** Meter accuracy.

Device for Measuring	Precision
K-type thermocouple	0.1 K
FHF04SC heat flux sensor	11 μV/(W/m^2^)
Vernier caliper	0.05 mm

**Table 2:** The statistics of the descriptive of the coefficient of thermal conductivity, λ, and the coefficient of thermal resistance, R, for samples made by 3D SLS wire printing technology, M—mean, SD—standard deviation, Me—median, Min—minimum, Max—maximum, K—kurtosis, Sk—skewness.

Variables	M	Me	Min	Max	SD	Sk	K
λ, W/m·K	0.053	0.056	0.035	0.067	0.011	0.313	1.402
R, (m^2^·K)/W	0.401	0.361	0.297	0.576	0.095	0.640	1.019

**Table 3:** The statistics of the descriptive of the coefficient of thermal conductivity, λ, and the coefficient of thermal resistance, R, and coefficient of heat transfer, U, for samples made by 3D DLP wire printing technology, M—mean, SD—standard deviation, Me—median, Min—minimum, Max—maximum, K—kurtosis, Sk—skewness.

Variables	M	Me	Min	Max	SD	Sk	K
λ, W/m·K	0.043	0.042	0.035	0.057	0.007	0.925	0.056
R, (m^2^·K)/W	0.948	0.951	0.680	1.155	0.131	0.463	0.542
U, W/m^2^·K	0.908	0.871	0.755	1.152	0.115	0.911	0.046

**Table 4:** Quantitative evaluation of main effects—identification of the impact of dominant and statistically significant inputs on the dependent variables (SS—sum-of-squares, df—degrees of freedom, MS—mean square, F—F ratio, *p*—significance level (*p*-values)).

Symbol That Identifies the Input Factors	SS	df	MS	F	*p*
λ (for SLS 3D printing)					
absolute term	0.1083	1	0.1082	10,828,269	0.00
number of air cells	0.0047	12	0.0004	39,169	0.00
error	2.59 × 10^−7^	26	9.99 × 10^−9^		
R (for SLS 3D printing)					
absolute term	6.2612	1	6.2612	364,463,623	0.00
number of air cells	0.3394	12	0.0282	1,646,517	0.00
error	4.46 × 10^−7^	26	1.71 × 10^−8^		
λ (for DLP 3D printing)					
absolute term	0.1128	1	0.1128	176,971.4	0.00
ε	0.0005	4	0.0001	175.8	0.00
n	0.0018	3	0.0006	923.8	0.00
ε × n	0.00024	12	0.00002	31.9	0.00
error	0.000026	40	0.000001		
R (for DLP 3D printing)					
absolute term	53.926	1	53.9260	293,821.5	0.00
ε	0.206	4	0.0516	280.9	0.00
n	0.732	3	0.2439	1328.7	0.00
ε × n	0.071	12	0.0059	31.9	0.00
error	0.007	40	0.0002		
U for the samples of the DLP 3D printing					
absolute term	49.4697	1	49.4697	531,265.3	0.00
ε	0.1457	4	0.0364	391.0	0.00
n	0.5489	3	0.1829	1965.0	0.00
ε × n	0.0817	12	0.0068	73.1	0.00
error	0.0037	40	0.00009		

## Data Availability

Data are contained within the article.
